# Unraveling substance abuse among Malawian street children: A qualitative exploration

**DOI:** 10.1371/journal.pone.0304353

**Published:** 2024-05-29

**Authors:** Lazarus Obed Livingstone Banda, Jane Thokozani Banda, Chigonjetso Victoria Banda, Eretia Mwaene, Ceasar Heatherwick Msiska

**Affiliations:** 1 School of Graduate Education, Beijing Institute of Technology, Beijing, China; 2 Higher Education, Ministry of Education, Malawi Government, Lilongwe, Malawi; 3 Political and Administrative Studies Department, University of Malawi, Zomba, Malawi; 4 Computer Studies, Nalikule College of Education, Lilongwe, Malawi; University of Botswana, BOTSWANA

## Abstract

This qualitative study adopts a phenomenological and symbolic interactionist approach to comprehensively explore substance abuse among street children in Lilongwe, Malawi. The research aims to uncover the complex sociocultural, economic, and environmental determinants influencing substance abuse within this marginalized cohort. Through in-depth semi-structured interviews, the study engages with street children to understand their subjective experiences, perceptions, and interpretations of substance abuse within their community context. Employing convenience, purposive, and snowball sampling strategies, the research collected data from street children, acknowledging their transient nature and societal challenges. Thematic analysis was conducted on interview transcripts to derive comprehensive insights. Results revealed five key thematic areas: familial absence and emotional void, societal normalization and peer pressure, economic hardships, coping mechanisms, environmental accessibility, and peer influence and belongingness. These themes highlighted the intricate interplay between personal experiences, socio-environmental factors, and peer dynamics, shaping the prevalence and persistence of substance abuse among street children. This study’s implications for practice underscore the need for tailored interventions and support mechanisms addressing substance abuse within this demographic. It emphasizes the urgency for context-specific strategies and policy formulations aimed at ameliorating the challenges faced by street children dealing with substance abuse in Malawi. Ultimately, this research contributes to a deeper understanding of substance abuse among marginalized street children, advocating for compassionate and contextually sensitive interventions within this overlooked drug abusers’ population subset.

## Introduction

In a sociocultural milieu marked by an escalating concern surrounding the pervasiveness of substance abuse among the marginalized kid’s cohort in Malawi, this study emerges as an earnest endeavor to delve into the labyrinthine intricacies of this prevalent issue. As street children, often relegated to the peripheries of societal attention, grapple with the profound impact of substance abuse within their lived environments, this inquiry seeks not merely to dissect but to comprehend the nuanced interplay between socio-contextual determinants and the realities of substance abuse. Within this backdrop, the study offers an illuminating narrative, delineating the contours of this complex phenomenon and shedding light on pathways toward tailored interventions and sustainable support mechanisms for this vulnerable demographic.

Subsequently, three specific objectives were derived from this aim. These were to (1) elucidate the intricate sociocultural, economic, and environmental determinants contributing to substance abuse among street children in Malawi; (2) conduct an in-depth exploration into the subjective experiences, perceptions, and interpretations of substance abuse among street children within their community context; and (3) delineate the multifaceted interplay between personal experiences, social interactions, and environmental factors shaping the prevalence and persistence of substance abuse among this cohort. To achieve these objectives, the following questions guided the study:

1. What sociocultural, economic, and environmental dynamics influence the emergence and perpetuation of substance abuse among street children in Malawi?
2. How do street children perceive, interpret, and attribute meaning to their encounters with substance abuse within their community setting?
3. What social interactions and environmental contexts significantly impact the prevalence and perpetuation of substance abuse among street children in Malawi?
4. In what ways can insights derived from the lived experiences of street children inform the development of effective interventions and tailored support systems addressing substance abuse within this demographic?

Previous scholarly endeavors investigating drug abuse in Malawi have predominantly centered on specific cohorts, notably adolescents [1, 2] and vulnerable adult populations [3–5] like commercial sex workers, farmers [6, 7], HIV/AIDS patients [8] and casual laborers [4]. However, the current study represents a notable departure from this established trend, meticulously focusing on an often marginalized and underrepresented segment within the societal framework. Specifically, this research endeavors to unravel the intricate complexities surrounding substance abuse among street children who fall within the primary school-going age demographic but exist outside the formal educational structure. This deliberate divergence in focus directs scholarly inquiry toward an often-neglected cohort, discerning the unique challenges and experiences encountered by street children entangled in drug-related issues, describing the uniqueness of this study, thereby contributing to a more comprehensive understanding of substance abuse within this distinct population subset.

Notwithstanding Malawi’s endorsement of the United Nations Conventions on Rights Child (UNCRC) on February 27, 1991, [9] there persists a rising demographic of unprotected street children dwelling within Malawi’s thoroughfares [10–12]. Importunate grievances persist regarding the disruptive impact of street youths on public order within Malawi. Unemployed street juveniles actively participate in aggressive street protests and instances of looting. Furthermore, the contemporary cohort of street youths has been linked to incidents involving robbery, the tragic fatalities of unassuming pedestrians in urban areas, and instances of pickpocketing. [10]. The rapid escalation of street youths assuming a formidable and concerning presence as a societal threat is a matter of apprehension. Current academic discourse underscores the disquieting prevalence of substance abuse among the youthful demographic in Malawi [13–15].

The correlation of these juveniles with the disruption of public order is evident. Incidents involving these individuals have been associated with acts of theft, alongside contributing to various forms of societal instability. However, the prevalent narrative in such coverage predominantly emphasizes the peril these youths pose rather than delving into the contextual circumstances they undergo. Notably, Nyasa Times’ September 25, 2021 publication titled “Street Kids Becoming a Terror in the City of Blantyre” (https://www.nyasatimes.com/street-kids-becoming-a-terror-in-city-of-blantyre/) and The Nation’s August 19, 2023 article echo this narrative.

Economically, the relationship between drug abuse and the health system in Malawi is complex and multifaceted [16]. Drug abuse poses significant health challenges, exerting pressure on the healthcare system [17], especially in ailing economies like Malawi. Substance abuse contributes to various health issues, including mental health disorders [16, 18], infectious diseases such as HIV/AIDS [3], injuries, and other medical complications. This increases the burden on healthcare facilities and resources [19]. Accessibility to substance abuse treatment and rehabilitation services might be limited in some areas of Malawi due to resource constraints within the health system [19]. This lack of access can exacerbate the health impacts of drug abuse, as individuals may not receive timely or adequate care.

This study holds paramount significance within the domain of substance and drug abuse research, notwithstanding the abundance of literature on this subject globally. Its salience lies in its focus on a distinct and marginalized cohort in its intent to unravel the intricacies of substance abuse within the specific geographical, sociocultural, economic, and environmental framework. The paucity of localized studies spotlighting the underrepresented demographic serves as a clarion call for attention to the plight of this vulnerable group. Subsequently, the nuanced exploration seeks to elucidate the multifaceted interplay between personal experiences, social interactions, and environmental influences, which distinctly shape the prevalence and sustenance of substance abuse within this cohort. This exploration is pivotal in understanding the contextual determinants and idiosyncrasies contributing to substance abuse among street children.

Furthermore, the study’s consequentiality extends beyond scholarly pursuit, offering pragmatic implications for intervention strategies and policy formulations. Insights gleaned from this nuanced inquiry into the lived experiences and perspectives of street children in Malawi could serve as a foundational platform for developing targeted interventions and tailored support mechanisms. These endeavors hold the potential for ameliorating the plight of street children dealing with substance abuse, necessitating culturally sensitive and context-specific interventions distinct from broader demographic approaches.

This study is not just an academic pursuit but a concerted effort to spotlight and address a critical societal issue within a specific and overlooked demographic. By offering a deeper understanding of substance abuse among street children in Malawi, it aspires to inform interventions, advocate for the marginalized, and contribute to a more holistic comprehension of this pervasive societal challenge.

### Literature review

The medicinal utility of a medicine is contingent upon its legal status and may vary [20]. The act of using a pharmaceutical substance to treat an ailment, mitigating the risk of a sickness, or enhancing overall well-being is sometimes referred to as “drug use.” However, when a substance is consumed for purposes unrelated to medical treatment, in quantities, potencies, frequencies, or methods that result in impairment of an individual’s physical or mental capabilities, it is classified as ‘substance abuse’ [20]. Various types of medicines and other legally available solvents have the potential to be subjected to misuse, including those that are prescribed for medical purposes.

Despite receiving much low attention [17], especially among the marginalized groups [21], [22, 23] substance use problems are on the rise not only in the US [24] but globally [25–29] as well as in many low-developed countries[23, 30] it is not all the members of the marginalized groups that develop substance abuse [31]. Drugs and medicine use can be induced by cultural, religious, and traditional medicinal use as in home remedies and economic pressures where the vulnerable and underprivileged service such drugs to cope with energy demands in their jobs [32] and to evade harsh realities of life [17, 33, 34]. Some kids live on the street because of homelessness, [35] have been overtaken by drug abuse. Lack of basic needs and parental guidance [35] in Nigeria, family financial pressures in Pakistan [36] social exclusion in Asians [37], social isolation in Delhi [38], short-term psychosocial and physiological gratification [39] peer influence in Gambia [40] catalyze drug and substance abuse among street kids.

Depression is a common challenge among the youths in Malawi, leading to adverse coping strategies [4, 13], with tobacco, alcohol, and marijuana being typical, among others [1, 3]. In alternative geographical locations such as India, the substances most frequently subject to misuse include alcohol, marijuana, heroin, bhang, hashish, different types of cough syrups, sedative pills, brown sugar, cocaine, tobacco [20], glue in Pakistan [36], and various solvents and inhalants in Bangladesh [39].

Street kids sometimes suffer rape [41], kidnapping, violence among themselves and from the community, police arrests, exploitation from the community, robbery, low immunity [41], and various other forms of abuse [36]. Despite adequate awareness about street life and associated dangers, the kids continue living this dangerous life because there are no viable options [36] live in cliques or gangs within which members protect each other against external forces and also take care of each other in many cases [36], and share the resources such as the drugs and substances.

However, the Malawian scenario has focussed much on the wrong things the kids do, neglecting what they also go through, thereby endangering their lives more. Locally, studies indicate a link between drug use and sexual risk behaviors among youths in Malawi [1, 3, 4]. The presence of ecological variables facilitated the engagement in hazardous behaviors through resource availability [3], the presence of supporting norms endorsed the view that drug use increased one’s capabilities [3], cultural endorsements [4], the absence of inhibitory factors, community-level poverty [4], interpersonal pressures, early initiation of drug use, and school dropout [4]. Other factors inducing drug abuse include accessibility to the resources [1]. Contrary to the professional teacher’s moral responsibility of supporting and enhancing retention [42] teachers’ sour relationships with learners induce school dropout [43–45], which is correlated with substance abuse [46, 47].

Individuals suffering from drug and substance abuse frequently have a range of accompanying health complications [4], including ailments such as pulmonary or cardiovascular disorders, cerebrovascular incidents, neoplastic growths, and psychiatric disorders [18], with detrimental consequences of prolonged substance abuse on the overall physiological well-being of the individual [18, 29]. Dealing with street kids through policing and strict law enforcement proved unpleasant in a study by Ribeiro [48].

### Theoretical framework

Various researchers have studied substance and drug abuse using different theoretical models [49], the Social Stress Model [22], and ecological models [4]. In contrast, others have narrowed down to a particular aspect of a model, such as Competence [50]. The theories are either biological (genetic or metabolic), psychological (inadequacy personality, self-derogation perspective, problem behavior proneness), sociological (routine activities, anomie, subcultural, social control, self-control, social disorganization, social learning, conflict theory) [49, 51, 52].

The theoretical framework guiding this study draws from phenomenology and symbolic interactionism. Within this context, phenomenology informs the qualitative inquiry by exploring participants’ subjective experiences and consciousness related to substance abuse. It focuses on understanding how street kids perceive and make sense of their world, shedding light on their lived realities concerning substance use.

In tandem, symbolic interactionism enriches the framework by spotlighting the significance of interactions, meanings, and symbols in shaping individuals’ behaviors and identities within their social contexts. This theoretical perspective underscores the importance of examining how street kids interpret and assign meaning to substance use within the framework of their social interactions and environments.

By integrating phenomenology and symbolic interactionism, this research framework aims to delve deeply into the subjective experiences and social dynamics surrounding substance abuse among street kids in Malawi, recognizing the interplay between personal experiences, interpretations, and social interactions within their lived environment.

## Materials and methods

### Design

This study was conducted in Malawi, a developing country [53] east of Zambia, south of Tanzania, and west of Mozambique [54, 55]. This study employs a qualitative research design centered around in-depth semi-structured interviews as the primary data collection method. The research design emphasizes a qualitative exploration, focusing on the richness and depth of narratives obtained through these interviews. By engaging street kids in Malawi through this method, the aim is to unearth nuanced perspectives, lived experiences, and personal insights related to substance abuse within their community. The qualitative nature of this design allows for a profound exploration of subjective experiences and contextual influences shaping substance abuse among street kids.

Aligned with an interpretivist research philosophy, the study recognizes and embraces the subjectivity inherent in individuals’ experiences and perceptions regarding substance abuse. The interpretivist philosophy underpins the belief that reality is socially constructed, emphasizing the importance of understanding the unique interpretations and meanings attributed by street kids to their experiences with substance abuse. This philosophy advocates for a qualitative approach that prioritizes exploring the complexities and intricacies of lived experiences, in line with the nature of in-depth semi-structured interviews.

### Instrument validity and reliability

The questionnaire designed for this study (S1 Appendix) underwent a rigorous content validation process, pilot testing, and subsequent analysis to ensure its effectiveness and reliability in capturing the intended information. Content validation constituted the initial phase of questionnaire refinement. A panel of subject matter experts, comprising individuals with substantial experience in the field pertinent to the study’s focus, meticulously scrutinized the questionnaire’s items. Their primary objective was to assess whether the questions comprehensively covered the targeted constructs and aligned with the study’s objectives. Through thorough discussions and evaluations, adjustments were made to refine the questionnaire, ensuring its relevance and accuracy in measuring the intended parameters.

A pilot test was conducted following content validation to gauge the questionnaire’s applicability and comprehensibility. A subset of the intended participant pool was administered the questionnaire, and their responses were assessed for clarity, coherence, and any ambiguities in the questions. Feedback from the pilot test participants facilitated the identification of potential issues, enabling necessary modifications to enhance the questionnaire’s readability and relevance.

Upon completion of data collection using the refined questionnaire, a meticulous analysis was undertaken to verify its reliability and efficacy.

A reliability test was conducted to assess the internal consistency and reliability of the interview guide, using Cronbach’s alpha coefficient, a commonly employed measure of internal consistency. The questionnaire items were analyzed for their correlation with each other to evaluate the overall reliability of the instrument. The Cronbach’s alpha coefficient for the interview guide was 0.78, indicating moderate internal consistency among the items. This coefficient suggests that the questionnaire items, when combined, demonstrate a satisfactory level of reliability in measuring the constructs related to societal, economic, and environmental dynamics, personal experiences, social interactions, interventions, and additional insights into substance abuse among street children in Malawi.

Additionally, the obtained responses were meticulously scrutinized to determine the questionnaire’s ability to capture the intended constructs accurately. The questionnaire’s validity and effectiveness in measuring the targeted parameters were affirmed through comprehensive analysis, ensuring its suitability for the study’s objectives.

This sequential process of content validation, pilot testing, and subsequent analysis fortified the questionnaire’s robustness and credibility, establishing its reliability in eliciting pertinent information aligned with the study’s focus.

### Data collection

Data collection commenced on July 12, 2023 and ended on October 13, 2023 and three sampling techniques were used: Convenient, purposive, and targeted snowball sampling.

Convenience sampling was employed to access street kids in easily reachable areas such as bustling markets, transportation hubs, and areas known for high street kid activity in Lilongwe, Zomba, and Blantyre. This approach aimed to engage with readily available participants who were likely to have experienced or observed the phenomenon [56–58] of substance abuse within their street communities. Researchers identified critical locations frequented by street kids based on local outreach programs and reports from social workers. Upon locating these areas, researchers approached street kids, explained the study’s purpose, and invited their participation.

A challenge was the potential bias in the sample due to accessibility issues. To mitigate this, efforts were made to diversify sampling locations and times, ensuring broader representation. The second challenge was that, initially, there was an apparent distrust among street kids. To mitigate this, rapport-building exercises were conducted to foster trust and comfort, involving interactions without immediately delving into the study.

Purposive sampling targeted street kids who had direct experiences or close exposure to substance abuse within their communities [59]. This approach aimed to capture nuanced and detailed insights from individuals familiar with the issue. With purposive sampling, social workers and community leaders familiar with street kids were consulted to identify individuals who could provide valuable insights. Participants with varying street life experiences and lengths were approached for interviews through referrals and recommendations.

However, data collectors experienced challenges related to limitations in sample size due to the specific criteria for participant selection. To address this, extensive networking and collaboration with local NGOs and community leaders were undertaken to widen the pool of potential participants. Besides, some initially identified participants were reluctant to engage due to apprehension or fear. To mitigate this, a gradual approach was adopted, emphasizing confidentiality and the non-judgmental nature of the study.

Due to its for its convenience and flexibility, especially useful in recruiting participants from hard-to-reach populations [60], snowball sampling was instrumental in reaching more isolated or less visible street kids who might not frequent typical gathering spots. It sought to leverage networks within the street community for referrals to individuals with diverse experiences. Seeds were established through convenient and purposive samples who were asked if they knew individuals not readily accessible in mainstream areas. These seeds were encouraged to refer peers or acquaintances who might have valuable insights [60] into substance abuse among street kids. Still more, the challenge was the limited number of referrals obtained. To address this, efforts were made to deepen relationships with initial contacts and foster a sense of trust to encourage more referrals. Besides, verifying information about the referred individuals’ experiences posed a challenge due to the nature of referrals. To mitigate this, triangulation of data from multiple sources and cross-referencing information with other participants’ narratives were employed to enhance reliability.

Moreover, continual adjustments and flexibility were exercised to accommodate the dynamic and transient nature of the street kid population in these regions. For referrals from the welfare personnel, getting adult consent from the people looking after them was easy.

### Data processing

#### Transcribing and translating data from Chichewa audio recordings to an English script

We began with choosing a reliable and secure offline storage medium. We used flash drives and backups to the research team leader’s email as attachments in case of data loss. No one was allowed to share any file with anyone not on the team. Ensuring the files were encrypted securely before backing them up to safeguard the confidentiality and integrity of the stored audio data. We then established a well-defined folder structure to categorize and organize the original Chichewa audio files: 1) Main folder: “Chichewa Audio Interviews.” 2) Sub-folders: “Interviews by Date,” “Interviews by Location, and by Participant Code.” We used clear naming conventions and employed consistent and descriptive file naming conventions for each audio recording in this way:

Naming format: YYYYMMDD_ParticipantCode_Location. For example: “20230215_LL1_Lilongwe”. Metadata and Documentation: We accompanied each audio file with metadata containing relevant information like interview date, participant details, location, and participant guarantor/guardian/parent/former school. We also maintained a separate spreadsheet indexing the audio files, including metadata, for easy reference and searchability.

#### Transcribing Chichewa audios to Chichewa text

The transcription process of Chichewa audio into written Chichewa text involved a systematic approach to accurately convert spoken content into a textual format while preserving linguistic nuances and expressions. Native speakers proficient in Chichewa were tasked with transcribing the audio recordings, ensuring precision and fidelity to the original spoken dialogues.

Initially, the transcribers familiarized themselves with the speech patterns, accents, and contextual nuances present in the Chichewa audio recordings through repeated listening sessions. This facilitated a comprehensive understanding of the spoken content before the transcription process. Following a verbatim approach, the transcribers meticulously transcribed the Chichewa speech into written text. Each audio segment was methodically transcribed, encompassing pauses, intonations, hesitations, and non-verbal cues. Specialized transcription software aided in playback and enabled careful consideration of specific sections during transcription.

Segmentation of the audio facilitated a systematic transcription process. The transcribers listened attentively to each segment, transcribing the Chichewa speech into written text, ensuring accuracy in capturing the spoken content phrase or sentence by sentence. To maintain accuracy and coherence, the transcribers thoroughly reviewed their work, rectifying any spelling errors, grammatical inconsistencies, or missing information in the Chichewa text. Consistent formatting and adherence to style guidelines ensured uniformity across the transcribed text. Transcribers relied on their understanding of the Chichewa language and cultural nuances to accurately represent the intended meanings expressed in the spoken content. This contextual understanding was essential for faithfully capturing the essence of the conversations in written form.

Validation of the transcribed Chichewa text against the original audio recordings ensured the accuracy and alignment of the transcriptions with the spoken content. This meticulous verification process guaranteed fidelity to the authentic dialogues. Upon completion, the compiled Chichewa transcripts were organized systematically, accompanied by metadata documenting transcription dates, transcriber details, and contextual information for future reference.

#### Translating from Chichewa text to English text

To accurately convey the meaning and essence of the original Chichewa content into English, the translation process from Chichewa to English involved a systematic approach. Proficient bilingual individuals skilled in Chichewa and English languages and with a good command of street slang were tasked with this translation process. Translators began by thoroughly understanding the context, nuances, and cultural references embedded within the Chichewa content. This initial comprehension phase was crucial in capturing the intended meanings and expressions conveyed in the Chichewa conversations. Using their bilingual proficiency, translators punctiliously rendered the Chichewa text into English, aiming to maintain the original content’s essence, tone, and context. The translation process aimed at conveying the meaning and intricacies of the Chichewa dialogue in English in a linguistically and culturally appropriate manner.

Reviewing and validating the translated English text was critical in ensuring accuracy and fidelity to the original Chichewa content. Multiple rounds of review by different translators or editors helped verify the coherence, accuracy, and alignment of the English translation with the original intent of the Chichewa conversations. Translators paid close attention to linguistic nuances, idiomatic expressions, street slang, and cultural references in the Chichewa content to accurately convey these elements in the English translation. This focus on cultural sensitivity and linguistic nuances aimed to preserve the richness and authenticity of the original content in the translated text.

#### The quality assurance and editing phase

The transcriptionists thoroughly proofread and revisited the transcribed Chichewa text to rectify any spelling errors, grammatical inconsistencies, or omissions. They carefully reviewed the text to ensure it accurately represented the spoken content, maintaining fidelity to the nuances, expressions, and context conveyed in the original audio recordings. Adherence to consistent formatting and style guidelines was ensured for uniformity and readability across the transcription.

In the case of translated English text, Translators conducted extensive reviews of the translated English text to validate linguistic accuracy and coherence. They checked for consistency in conveying the intended meanings, idiomatic expressions, and cultural nuances from the original Chichewa content into English. The review process involved multiple rounds of editing to refine the translation, rectifying any mistranslations, ambiguities, or discrepancies between the Chichewa and English texts.

During the quality assurance phase, emphasis was placed on ensuring the accuracy of content while retaining the essence and contextual relevance of the original material. Cross-referencing between the transcribed or translated text and the original audio recordings or Chichewa source text was performed to validate alignment and accuracy. Attention was given to maintaining linguistic coherence, cultural sensitivity, and contextual appropriateness in the transcribed or translated content. Any inconsistencies or ambiguities identified during the review process were addressed and rectified to enhance the overall quality and accuracy of the final transcriptions or translations. Ultimately, the quality assurance and editing phase played a pivotal role in refining and validating the transcribed Chichewa text or translated English text, ensuring that the final output accurately represented the original content with precision, clarity, and linguistic fidelity.

#### Data verification and validation

To begin with, for transcribed Chichewa text, we conducted a verification and validation process to confirm the accuracy, consistency, and contextual fidelity of the transcribed Chichewa text or translated English text, thereby enhancing the reliability and trustworthiness of the final transcriptions or translations. Transcribers cross-referenced the transcribed Chichewa text with the original audio recordings. This verification step ensured that the written content accurately reflected the spoken words, including nuances, intonations, and pauses in the audio. After that, the transcribers and reviewers considered the context of the conversations within the Chichewa text. This involved verifying that the transcription captured the literal words and the underlying meanings and intentions conveyed during the conversations.

Secondly, for translated English text, Translators cross-checked the translated English text against the original Chichewa content. This process aimed to validate the accuracy of the translation by ensuring that it conveyed the intended meanings and nuances present in the Chichewa source material. Translators and reviewers verified the translated English text for cultural sensitivity, ensuring that idiomatic expressions, cultural references, and contextual nuances from the Chichewa content were accurately conveyed in English.

Besides, seeking input and validation from individuals proficient in Chichewa and English languages ensured accuracy and authenticity in the transcribed or translated content. Multiple reviewers or experts collaboratively examined the transcribed Chichewa text or translated English text to validate and confirm the accuracy of the content. Comparing and corroborating information across multiple transcripts or translations helped cross-reference data, ensuring consistency and reliability across the collected data sets.

### Ethical standards

We obtained ethical approval from a Research Ethics Committee with approval number Ref No: MZUNIREC/DOR/22/55. We highly regarded ethical considerations in this study since it involved minor participants, especially those staying away from their parents or guardians. Informed consent procedures were followed, ensuring the minors and their guardians were informed about the study’s purpose, procedures, risks, and benefits in age-appropriate language.

Assent: The minor’s agreement to participate, was obtained if they could understand the study, while parental or guardian consent was sought. Guardianship for the participants fell into three categories: Some kids stayed with their parents on the streets. In that case, such parents consented to the study. Others were under the care of non-governmental organizations, while the rest were free rangers who either told us how to find their parents in the villages, or could not trace them.

We ensured strict confidentiality measures to protect the identity and privacy of the minor participants. Personal information was kept confidential and shared only among authorized personnel involved in the study. Emphasis was placed on respecting minors’ autonomy to participate or withdraw from the study without coercion or pressure. Measures were taken to ensure that participation was voluntary and that juveniles were not unduly influenced or compelled to participate in the research. Some minors were staying away from their parents or guardians, so extra sensitivity was exercised to ensure their safety, well-being, and emotional comfort during interviews or interactions.

The study adhered to legal requirements and institutional guidelines regarding research involving minors, ensuring compliance with ethical standards and regulations. Collaboration with local NGOs, community leaders, and relevant authorities helped navigate the ethical complexities of involving children living away from their parents. It ensured that their interests and well-being were prioritized. For all the participants whose parents were also living on the streets, it was much easier to get consent to interview their kids, just like those kids under the custody of an organization. However, such parents usually demanded some money before we could interview their kids. This was understandable since the parents were also very destitute on the streets. On the contrary, some street kids stay far away from their homes or parents. For those kids who could help us find their original homes or schools attended before they lived on the streets, we traced their parents either directly or through their previous schools for consent. Due to the complexity of tracing their villages, the District Commissioners helped trace the traditional leaders who led us to the parents/guardians’ homes. The kids whose guardians were minors or whose parents or guardians could not be traced were not interviewed.

### Data analysis

The qualitative data collected from in-depth, semi-structured interviews with street children in Malawi underwent a meticulous thematic analysis to extract and elucidate thematic patterns within their sociocultural context. This analytical journey commenced with a deep immersion into the raw textual data from interview transcriptions. Researchers extensively engaged in repeated readings and familiarization exercises, cultivating a profound understanding of the narratives that conveyed the substance abuse experiences articulated by street children.

The subsequent step involved systematically segmenting data into meaningful units through initial coding. These units encapsulated substantive elements related to substance abuse experiences, perceptions, and socio-contextual nuances expressed by the street children. Through a rigorous thematic coding process, data segments were grouped, categorized, and organized into preliminary themes. Iterative refinement and revision were vital, ensuring comprehensive coverage of diverse substance abuse narratives capturing the essence of experiences, interpretations, and socio-contextual influences.

Themes underwent critical review, refinement, and precise definition to ensure coherence, relevance, and distinctiveness. Careful scrutiny of thematic content against the original data substantiated interpretations and ensured alignment with the substance abuse experiences conveyed by the street children. Subsequently, a hierarchical coding structure was formulated, organizing the refined themes into a coherent framework. This structured representation illustrated interrelationships and subordination among themes, establishing a comprehensive and systematic architecture for interpreting substance abuse narratives.

Each refined theme underwent comprehensive elaboration, including detailed descriptions and direct data exemplifications. This process contextualized and substantiated thematic content, firmly anchoring interpretations within the substance abuse experiences narrated by the street children. The finalization and integration of themes resulted in a cohesive narrative that reflected the rich tapestry of substance abuse experiences among street children in Malawi. This thematic map encapsulated diverse dimensions, nuances, and salient aspects of substance abuse narratives.

Table 1 is an overview of the demographic characteristics of this qualitative study’s participants. It provides a detailed portrayal of the diverse socio-demographic attributes, offering a nuanced understanding of the contextual backdrop against which substance abuse experiences among street children were explored.

**Table 1:** An overview of the demographic characteristics of the participants.

Kid	Age	Education level	How I started drug use	Drug often used	street residence	Crimes ever involved in	Reason to be in the street
LL1	10	Std 2	My friends enticed me	Marijuana, Bostic, glue, paraffin	3 years	Robbery, stealing	Parents shouting at me
LL2	14	never		alcohol, Marijuana, glue	14	Fighting, stealing, robbery	Born in the street
NU1	13	3	To fit in	Marijuana, alcohol, paint	6	Stealing, robbery	Teacher flogged me,
NU2	15	3	Protection	Marijuana, alcohol, Bostic	5	Fighting, stealing, robbery	Teacher mocking me
BT4	16	4	Comfort, emotional security, acceptance	Alcohol, paraffin, glue, marijuana	Not sure	Fighting, stealing, robbery	Parents always blamed me for anything
MZ5	16	3	Acceptance and sharing	Marijuana, alcohol	Not sure	Stealing	A choir leader refused to let me play the church piano.
MZ6	13	2					
8	15	never	No home, mother introduced it to me	Marijuana, Bostic, glue, alcohol	15	Stealing, fighting, robbery	Born in the street
LL5	Not sure	Never	Born in street	Marijuana, Bostic, glue, alcohol	Not aware	Stealing, fighting, robbery	Born in the street
LL7	Not aware	Never	Born in street	Marijuana, glue	Not sure	Stealing, fighting, robbery	Born in the street
NU10	15	Never	Born in street	Alcohol, glue	15	Stealing, fighting, robbery	Born in the street
MZ12	15	6	Hunger: my father used to deny me food once I failed homework	Alcohol, glue, marijuana, paraffin, paint	3	Stealing, fighting, robbery	Dad whipped me too much because I was watching TV instead of doing homework
ND3	16	3	Friends seemed happier	glue, marijuana, paraffin,	4	Rape, robbery	My parents were never at home
ND8	12	2	Needed belonging	Marijuana, Bostic, glue, alcohol	3	Fighting, stealing,	I was lonely
TK2	15	4	Wanted companionship	glue, marijuana, paraffin,	5	Robbery, stealing, rape	My father never talked to me

The demographic descriptions encompass various sociocultural, economic, and contextual facets, including age, educational background, and length of street residency, as pertinent socio-economic indicators. These descriptors serve as foundational elements in contextualizing the narratives and experiences shared by the participants, shedding light on the intersectionality of their backgrounds and the dynamics influencing substance abuse within their lived environments.

The table outlines the profiles of fifteen street children engaged in substance abuse in Lilongwe, Zomba, and Blantyre. The participants’ ages range from 10 to 16 years old. Education levels vary from never attending school to reaching grade 6. The initiation into drug use varied, with influences such as peer pressure, fitting in, protection, comfort, and parental influence.

Commonly used substances include marijuana, glue, alcohol, bostic, paraffin, and paint. The duration of street residence varies, with some born into street life. Participants were often involved in crimes like stealing, fighting, robbery, and, in some cases, more severe offenses like rape.

Reasons for being on the street varied, including parental issues like shouting or blaming, rejection from social groups, feeling born into street life, seeking emotional security, and needing companionship or belonging. These street children had diverse backgrounds but shared a commonality in experiencing neglect, abuse, or emotional distress, leading them to seek solace in substance abuse and street life.

This comprehensive demographic overview enriches the study’s contextual understanding and lays the groundwork for comprehending the diverse perspectives and experiences elucidated through qualitative inquiry. By delineating the socio-demographic characteristics of the participants, this table contributes to a more holistic comprehension of substance abuse experiences among street children in Malawi within the broader sociocultural framework.

## Results and discussion

### Phenomenological lens

Through their narratives, a profound sense of detachment and yearning for connections echoes persistently. Expressions like “Using drugs makes us feel better, even if it’s just for a while” delineate their pursuit of fleeting solace amidst their profound desolation. The name “Jamaica” likely holds symbolic significance for these street kids. Jamaica is often associated with reggae music, Rastafarian culture, and a relaxed, carefree atmosphere. By naming their area after Jamaica, these kids might be creating a symbolic link to a place or culture known for its laid-back attitude and perhaps the association with marijuana.

Furthermore, their use of glue or Bostic might reflect a deeper socio-economic context, signifying a coping mechanism or an escape from difficult circumstances, possibly stemming from poverty, neglect, or other challenging life situations.

### Symbolic interactionist lens

Peer influence and the societal normalization of drug use, encapsulated in statements such as “Some older kids here said it would help forget the hunger and the cold, so I tried it,” underscore the symbolic significance attached to drug use as a coping mechanism within their social spheres. This nomenclature, “Jamaica,” holds symbolic significance within the street children’s social interactions and shared meanings beyond its literal geographical reference. Symbolic interactionism, as a theoretical framework, emphasizes the role of symbols, language, and shared meanings in shaping human behavior and social interactions.

It could symbolize a perceived connection or similarity to a place associated with marijuana or substance culture. This symbolic renaming reflects how these children construct and share meanings within their social circles. This renaming could also serve as a form of coded language within their group, establishing a collective identity, besides reflecting the aspirational or escapist aspects of their substance use, and mirroring some cultural references often associated with the Caribbean nation of Jamaica.

### Thematic analysis

The narratives portray various origins of early drug exposure, ranging from parental influence (using substances to sedate infants or alleviate hunger) to environmental factors (community survival via substance abuse) and abandonment, such as children born to sex workers and left homeless.

#### Theme 1: Familial absence and emotional void

The absence of familial structures surfaces prominently in their narratives, evoking sentiments of isolation and dearth of guidance. Expressions like “No families, no schools. So, we end up finding comfort in drugs” portray a stark emotional void. Substance use serves as a coping mechanism, momentarily alleviating their profound solitude. The following quotes substantiate this experiential terrain:

“There’s no one to guide us. No families, no schools. So, we end up finding comfort in drugs.”

“We feel alone. Using drugs makes us feel better, even if it’s just for a while.”

These results corroborate prior studies [35, 37, 38].

#### Theme 2: Normalization and peer pressure

Societal normalization of substance use and peer pressures significantly mold their initiation into drug use. The perception of drug use as commonplace, as reflected in the sentiment “It’s like a normal thing around here, …,” delineates the sway of societal norms. Furthermore, the quest for acceptance and inclusion, articulated in “Everyone else was doing it, …,” underscores the influence of peers in shaping substance initiation corroborating Bah’s study [40]. Through the phenomenological lens, we discover that the kids learn this based on their everyday lives. The physical and social environments these kids are exposed to play a vital role in their unhealthy behaviors. The following quotations emphasize this contextual backdrop:

“We see adults doing it too. It’s normal around here, so we try it too.”

“Everyone else was doing it, and I didn’t want to feel left out. So, I tried it, too.”

#### Theme 3: Challenges met on the street

The participants indicated knowledge about how this illicit substance usage impairs their health. They revealed that substance abuse leads to cognitive impairment, physical ailments, social ostracization, distrust, and involvement in criminal activities. However, these challenges are perceived as part of their reality, leading to a sense of resignation and adaptation to this lifestyle. Limited access to healthcare due to societal prejudice, financial constraints, and logistical difficulties exacerbates their health issues, prompting reliance on informal or intermittent healthcare support.

#### Theme 4: Economic hardships and coping mechanisms

Economic adversities, notably cold, hunger, destitution, and the bleak outlook for the future, emerge as pivotal triggers propelling substance engagement, driving these children towards substance use as a means of escape. Quotations substantiating these experiential accounts include:

“When you’re hungry, and there’s no one to help, drugs make you forget about the pain in your stomach.”

“Sometimes it’s boredom. There’s nothing to do, and drugs seem like a way to pass the time.”

Consistent with similar studies, these results indicate that the drugs are viewed as a coping mechanism to alleviate physical and emotional distress caused by poverty [35, 38].

Expressions like “Drugs make you forget about the pain in your stomach” accentuate how substances offer a temporary respite from dire economic circumstances, just as reported by a similar study in Dhaka [39], in which short-term gratification was used to survive the physiological pains. Moreover, the narrative of using drugs to combat boredom reflects substance use as a diversion from the bleak monotony of their lives. Similarly, other studies report the same motivations [61, 62]. The existential vacuum is positively related to drug and substance abuse [62].

#### Theme 5: Environmental accessibility and peer influence

The environment, steeped in accessibility to drugs, becomes an intrinsic part of their everyday existence. Testimonies depict the ubiquity of drugs and the dearth of constructive alternatives, underscoring the environment’s role in perpetuating substance reliance. From the symbolic interactionism lens, one sees the kids using figurative language in which they substitute the original name of the location where they find the drugs (Falls Area) with a symbolic name (Jamaica), suggesting how common and easily accessible marijuana is within their environment, echoing the common belief that smoking marijuana is legal in Jamaica, contrary to the legal frameworks. Many of the street kids identify themselves with Rastafarianism than any other religion in Malawi. The act of possessing quantities above 56 grams of marijuana continues to be classified as a criminal offense, subjecting individuals to potential criminal charges and subsequent legal proceedings. The consumption and possession of cannabis is permitted for those who identify as Rastafarians since it is recognized as a religious practice. Rastafarians are allowed to utilize marijuana for religious purposes [63]. Just as Echoes from the narratives:

“It’s everywhere. You can find it easily, so it becomes a part of everyday life.”

“There’s not much to do here, and it’s easy to get stuff.”

#### Theme 6: Peer influence and belongingness

It’s also seen as a means of camaraderie within peer groups and a method to navigate the harsh realities of street life. Everyone considers belongingness a basic need [Abraham Maslow’s hierarchy of needs]. Social exclusion [37], homelessness [35], isolation [38] from schools, and absence of parental figures create a vacuum that gets filled in the street. Belonging to a figure of influence among street kids was seen as a way to ensure security. Such authority is taken seriously among their cliques, and advice from older kids is “virtuous” without perceived autonomy and alternative relatedness. They obey what elders command them for protection from robbery and bullying from others. One kid said:

“Sometimes, fellow members can rob you, so you need protection from an elder who tells you to practice what he does.” Our findings echoe what Molahlehi reports [41].

Apart from perceiving substance abuse as a temporary respite from loneliness, boredom, stress, and societal neglect, peer dynamics wield a formidable influence on the initiation of drug use. Older street children serve as influential figures, driving the narrative of substance experimentation to seek companionship and a sense of belonging. Similar sentiments were reported by Bah [40]. Articulations from the children:

“Some older kids here said it would help forget the hunger and the cold, so I tried it.”

“I saw others sniffing something to feel better when they were sad. I wanted to feel better, too.”

While drugs foster a sense of belonging, they also spur conflicts among peers. Despite this, there’s a protective aspect within these groups, showcasing a paradoxical mix of support and conflict. The following quotations illustrate these findings, consistent with a similar study in Pakistan [36]:

“When I’m high, I easily get irritated and fight over trivia, ending up being hurt or injuring others.”

“When you’re high, you feel like you can overpower anyone. In case they’ve anything you wanna them share with you, and they dare not give some to you, certainly you feel like bashing their heads against the walls.”

“When you belong to one of these gangs and smoke or drink what the leaders tell you, you are assured of being supported in case members from conflicting gangs abuse or provoke you.”

“Sometimes, as you walk on the streets, some guys will want to rob you, but if they know your gang, they will be scared.”

#### Theme 7: Vengeance and common crimes under the influence of substance abuse

The discourse among the adolescent demographic under scrutiny reveals a distressing pattern wherein these individuals are implicated in severe criminal activities, often under the influence of intoxicants or as a means to procure funds for the acquisition of illicit substances. Notably young, these individuals confessed involvement in heinous crimes encompassing murder, armed robbery, and utilizing crude weapons like knives and bicycle spokes to coerce victims into relinquishing personal belongings such as purses, handbags, and mobile devices. Consistent with Molahlehi’s findings, this study reaffirms that some children turn to robbery as a manifestation of anger stemming from being preyed upon by peers who were entrusted with the role of safeguarding them [41]. Additionally, reports surfaced indicating participation in acts of rape, purportedly as retribution for assaults inflicted upon their cohort by external perpetrators. Instances of theft and physical altercations over trivial disputes were also disclosed.

These revelations align with existing scholarly investigations conducted in various global contexts [41]. These juveniles disclosed that engagement in these transgressions did not stem from inherent malevolence but rather as a response to perceived societal condemnation and mistreatment. They emphasized discriminatory attitudes exhibited by the broader public and medical professionals, significantly influencing the hostile reprisal they enacted against anyone they targeted. These findings corroborate existing research from diverse international studies [35, 40, 41].

Despite the community feeling that the kids are inherently wrong, it was intriguing to learn that they lamented vulnerability to exploitation by adults offering free drugs or coercing them into criminal activities, highlighting the need for protective measures and empathetic understanding rather than condemnation. Molahlehi also discovered that there is a considerable level of unreported exploitation of street kids by community members and the police [41]. While not all street children engage in criminal activities, environmental pressures, societal prejudices, and self-preservation instincts sometimes lead to their involvement in crimes, often for survival or protection.

#### Theme 8: Interventions and support systems

The proposed interventions outlined by the children exhibited a proactive and constructive approach. These encompassed community engagement, parental reinforcement, access to education, ongoing dialogue, and peer mentorship initiatives to mitigate substance abuse while offering alternative avenues. These suggestions underscored a dichotomy wherein, despite potential reluctance from some parents to reintegrate these street children, a yearning for a sense of belonging within a home environment persists among certain youths. Arrangements were made for the skeptical parents whose kids desired the reunion to psychologists to navigate a compromise for a smooth reinstatement transition into their homes. Psychologists were responsible for counselling both sides while local youth organizations were to be identified to integrate the kids appropriately into the society. However, follow up to this arrangement was beyond the scope of our study.

While disillusionment with formal education emerged due to experiences of mistreatment by teachers, parental pressure concerning homework, and peer-related conflicts, a segment of these children still harbored a strong desire for learning opportunities. Interestingly, amidst these perspectives, only one street child mentioned cessation of substance abuse. Notably absent from the proposed solutions was rounding up these children. This absence reflects a prevailing negative sentiment towards policing strategies, consistent with findings observed in analogous studies [41, 48].

### Conclusion

In culmination, this study embarked on a painstaking qualitative exploration, delving into the multifaceted realm of substance abuse experiences among street children in Malawi. Through in-depth, semi-structured interviews, a comprehensive understanding of the intricate sociocultural, economic, and environmental dimensions influencing substance abuse within this marginalized cohort was meticulously unraveled.

The narrative analysis illuminated a diverse tapestry of substance abuse narratives intricately interwoven with the lived experiences, perceptions, and interpretations of street children. Themes encapsulated the nuanced interplay between personal encounters, socio-environmental dynamics, and the broader fabric of societal influences, providing a comprehensive mosaic of substance abuse realities.

The thematic analysis culminated in a coherent framework that unveiled the complexities of substance abuse experiences and illuminated pathways for tailored interventions and support mechanisms. These findings hold profound implications for policy formulation and targeted interventions, advocating for context-specific strategies distinct from broader demographic approaches.

Moreover, the study’s significance resonates beyond academic spheres, serving as a clarion call to address the plight of marginalized street children grappling with substance abuse issues. By amplifying their voices and experiences, this study advocates for inclusive interventions and support mechanisms tailored to their specific needs, fostering a more holistic approach to addressing substance abuse within this overlooked demographic.

In essence, this qualitative inquiry stands as a testament to the imperative of understanding substance abuse through the lens of the lived experiences of street children. The insights gleaned not only contribute to scholarly discourse but also advocate for tangible societal change, emphasizing the urgency for compassionate and contextually sensitive interventions to ameliorate the plight of street children confronting substance abuse in Malawi.

A localized follow-up investigation could pivot towards the assessment and implementation of bespoke intervention initiatives explicitly tailored for this demographic, concentrating on evaluating the efficacy and application of community-driven intervention strategies designed to mitigate substance abuse issues among street children. Augmenting quantitative assessments with qualitative inquiries, this research would navigate the nuanced landscape of participants’ experiences, eliciting firsthand perspectives to elucidate the elements contributing to the success or limitations of these interventions, thus offering a robust blueprint for tailored intervention strategies to address substance abuse among street children in Malawi.

## Supporting information

S1 File Data script. (PDF)

S1 Appendix Interview questionnaire. (DOCX)
