# Supplementary material for: Unraveling substance abuse among Malawian street children: A qualitative exploration
Source: PLoS One. 2024 May 29;19(5):e0304353. doi: 10.1371/journal.pone.0304353 (PMC11135666; doi:10.1371/journal.pone.0304353)
Supplement: S1 File — (PDF) [file pone.0304353.s001.pdf]

Data script

Interview Transcript: In-Depth Interview with 14 Malawian Street Kids on Substance Abuse

Interviewer: Let's start with a little about you. Can you tell me your age, how long you've been on the streets, something about your family, and your education level?

Child 1: I'm 15. Been on the streets for about 5 years now. I don't really have any family around, never really went to school either.

Interviewer: What circumstances led you to live on the streets?

Child 2: It was mainly hunger and no one to take care of us at home. School wasn't an option either. No families, no schools. So, we end up finding comfort in drugs.

Interviewer: What substances are commonly used among street children here?

Child 3: Mostly sniffing glue, smoking weed, and sometimes cheap alcohol. Marijuana is pretty common around the place we call Jamaica, it’s easy to get there.

Interviewer: How did you first become aware of substance abuse among street children?

Child 4: I saw others sniffing something to feel better when they were sad. I wanted to feel better, too.

Interviewer: What do you think leads street children to start using these substances?

Child 5: We feel alone. Using drugs makes us feel better, even if it’s just for a while.

Interviewer: How does the community view substance abuse among street children?

Child 6: It’s like a normal thing around here. People see adults doing it too, so we try it too.

Interviewer: How do cultural practices or beliefs influence substance use among kids?

Child 7: Everyone else was doing it, and I didn't want to feel left out. So, I tried it, too.

Interviewer: In your experience, how do daily economic activities relate to substance abuse among street children?

Child 8: When you’re hungry, and there’s no one to help, drugs make you forget about the pain in your stomach.

Interviewer: What role do you think the environment plays in substance abuse?

Child 9: There’s not much to do here, and it’s easy to get stuff from Jamaica. It becomes a part of everyday life.

Interviewer: If comfortable, can you share any personal experiences related to substance use?

Child 10: Sometimes it’s boredom. There’s nothing to do, and drugs from Jamaica seem like a way to pass the time.

Interviewer: How do you think substance abuse affects the lives of street children?

Child 11: It's tough, you know. Sometimes, fellow members can rob you, so you need protection from an elder who tells you to practice what he does.

Interviewer: What kind of support do street children who use substances need?

Child 12: Some older kids here said it would help forget the hunger and the cold, so I tried it.

Interviewer: Are there existing interventions or support systems for substance abuse among street children in your area?

Child 13: Not much really. It’s hard to find real help.

Interviewer: What do you think would be an effective way to address substance abuse among street children?

Child 14: We need real places to stay and real schools, not just being chased around. We need someone to guide us.

Interviewer: How can insights from street children be used to improve support and interventions?

Child 1: Listen to us; we know what we need. We're not just on the streets because we want to be.

Interviewer: Is there anything else you would like to share about your experiences or opinions on this topic?

Child 2: Just that, it's tough out here. People think we do drugs just for fun, but it's not like that. It's about surviving every day.

Interviewer: Thank you all for sharing your experiences and thoughts. Your insights are invaluable in understanding and hopefully improving the situation.
