# Supplementary material for: Unraveling substance abuse among Malawian street children: A qualitative exploration
Source: PLoS One. 2024 May 29;19(5):e0304353. doi: 10.1371/journal.pone.0304353 (PMC11135666; doi:10.1371/journal.pone.0304353)
Supplement: S1 Appendix — (DOCX) [file pone.0304353.s002.docx]

**Appendix: Interview Questionnaire**

**Semi-structured in-depth interview questionnaire guide with street kids**

**Section 1: Background information**

1. Can you tell me a bit about yourself? (age, time spent on the street, biological family status, education level, etc.)
2. What circumstances led you to live on the streets?

**Section 2: Perception and understanding of substance abuse**

1. What substances are commonly used among street children in this area?
2. How did you first become aware of substance abuse among street children?
3. What do you think leads street children to start using these substances?

**Section 3: Sociocultural and economic factors**

1. What do you think people you meet view you as someone who indulges in substance abuse in the street?
2. How do cultural practices or beliefs influence you to begin or continue indulging in substance use?
3. In your experience, how do daily economic activities impact your indulgence in substance abuse?

**Section 4: Environmental influences**

1. What role do you think the environment plays in substance abuse? (e.g., availability of substances, price, treatment from people, peer influences)

**Section 5: Personal experiences and perspectives**

1. If comfortable, can you share any personal experiences related to substance use?
2. How do you think substance abuse affects the lives of street children?
3. What kind of support do street children who use substances need?

**Section 6: Intervention and support**

1. Are there existing interventions or support systems for substance abuse among street children in your area?
2. What do you think would be an effective way to address substance abuse among street children?
3. How can insights from street children be used to improve support and interventions?

**Closing questions [with thanks].**

1. Is there anything else you would like to share about your experiences or opinions on this topic?
